# Nature-Inspired Biomolecular Corona Based on Poly(caffeic acid) as a Low Potential and Time-Stable Glucose Biosensor

**DOI:** 10.3390/molecules28217281

**Published:** 2023-10-26

**Authors:** Maria Kuznowicz, Artur Jędrzak, Teofil Jesionowski

**Affiliations:** Faculty of Chemical Technology, Institute of Chemical Technology and Engineering, Poznan University of Technology, Berdychowo 4, PL-60965 Poznan, Poland; maria.m.kuznowicz@doctorate.put.poznan.pl

**Keywords:** glucose biosensor, poly(caffeic acid), electrochemical biosensor, real samples, glucose detection

## Abstract

Herein, we present a novel biosensor based on nature-inspired poly(caffeic acid) (PCA) grafted to magnetite (Fe_3_O_4_) nanoparticles with glucose oxidase (GOx) from *Aspergillus niger* via adsorption technique. The biomolecular corona was applied to the fabrication of a biosensor system with a screen-printed electrode (SPE). The obtained results indicated the operation of the system at a low potential (0.1 V). Then, amperometric measurements were performed to optimize conditions like various pH and temperatures. The SPE/Fe_3_O_4_@PCA-GOx biosensor presented a linear range from 0.05 mM to 25.0 mM, with a sensitivity of 1198.0 μA mM^−1^ cm^−2^ and a limit of detection of 5.23 μM, which was compared to other biosensors presented in the literature. The proposed system was selective towards various interferents (maltose, saccharose, fructose, L-cysteine, uric acid, dopamine and ascorbic acid) and shows high recovery in relation to tests on real samples, up to 10 months of work stability. Moreover, the Fe_3_O_4_@PCA-GOx biomolecular corona has been characterized using various techniques such as Fourier transform infrared spectroscopy (FTIR), high-resolution transmission electron microscopy (HRTEM), atomic force microscopy (AFM), X-ray photoelectron spectroscopy (XPS), and Bradford assay.

## 1. Introduction

Diabetes currently belongs to the civilization diseases [1]. According to the World Health Organization (WHO), about 5% of the world’s population suffers from this illness, which is growing yearly [2]. Counteracting its development and impact on human life includes frequent glucose measurements, which are necessary to maintain normal sugar levels. In addition, it necessitates continuous development and the need to acquire new glucose biosensors [2].

One of the critical parameters for those fighting diabetes is maintaining a constant glucose concentration at the appropriate level, which allows for avoiding health complications. However, since maintaining a constant blood glucose parameter is complex, multiple daily measurements are necessary [3,4]. The following are general blood glucose ranges for adults without diabetes: (i) normal is less than 100 mg/dL (5.6 mM/L); (ii) prediabetes from 100 to 125 mg/dL (5.6 to 6.9 mM/L); (iii) diabetes is 126 mg/dL (7 mM/L) or higher. Therefore, it is crucial to produce highly stable, sensitive, and selective biosensors that enable accurate and quick tests at relatively low cost [4].

Many techniques are on the market for analyzing blood glucose levels, but the most extensively tested are optical and electrochemical methods [5]. In particular, amperometric methods and enzyme biosensors have gained popularity in personal glucose monitoring and especially in point-of-care testing (POCT) [4,6]. Several detailed studies were carried out in the literature to obtain new hybrid nanomaterials that would allow the creation of improved electrode surface structures to obtain improved detectors with more sensitive and selective measurements with long-term stability [7,8]. 

Poly(caffeic acid) in its structure contains oxidized and reduced o-hydroquinone/o-quinone pairs, making it possible to use this compound in electrocatalytic processes [9]. Previous studies using PCA for sensor applications have shown that the present redox pair characterizes a two-electron PCA reaction process [10]. These studies showed that the advantage of PCA as an electrode material is the o-quinone redox probe that facilitates the electron transfer reaction between the target analyte and the electrode. In addition, the electrodes thus modified and obtained revealed a commensurate electrocatalytic current and peak potential [11]. 

Research conducted by Li et al. allowed the construction of a PCA-based electrochemical sensor for the detection of acetaminophen. The use of PCA for this purpose allowed the detection of this compound at a lower potential (0.33 V) than in the case of the GC bare electrode (0.415 V) [11]. In the case of the detection of chlorine by Kesavan et al. based on the GC/PCA@ERGO electrode, well-defined anodic and cathodic peaks were observed at 0.21 and 0.17 V, respectively, compared to the bare electrode [10]. The mechanism of the catalytic reaction of catechol, which is electrochemically oxidized by electrons from the electrode, and as a result the amperometric current can be monitored, has also been investigated by Lee et al. Their research presents the mechanism and electrochemical detection of glutathione [12]. Compounds like dopamine (DA) and ascorbic acid (AA) can be identified using electrochemical techniques based on anodic oxidation. However, a significant issue is that the oxidation potentials for AA and DA almost always occur at the same potential and overlapping voltammetric responses. In work presented by Li et al., a clear separation of oxidation peaks of AA and DA was presented, indicating that PCA coverage facilitated the simultaneous detection of AA and DA at different potentials [13].

Moreover, detection in the lower potential can reduce the contribution of interfering agents to the overall background current, making it easier to distinguish and measure the signals related to the target substances. This reduction in background currents improves the signal-to-noise ratio and enhances the sensitivity of the measurements [14]. Moreover, detection in the lower potential can reduce the contribution of interfering agents to the overall background current, making it easier to distinguish and measure the signals related to the target substances. This reduction in background currents improves the signal-to-noise ratio and enhances the sensitivity of the measurements. The low influence of interfering agents is important in the case of medical or environmental research, where many other compounds can be found in addition to the tested compound, which can significantly affect the final measurements [15,16].

In this work, a nature-inspired poly(caffeic acid)@magnetite nanomaterial was obtained. The component’s properties, like the magnetic stability and biocompatibility of magnetite, as well as the functionality and ability to transfer electrons through PCA, enabled the creation of a novel hybrid material with synergistic properties. The proposed system was linked with glucose oxidase (GOx) to form a biomolecular corona nanostructure. This material was used to modify the screen-printed electrode (SPE) and was used for electrochemical tests to detect glucose in real solutions. The novelty of the work unveils the new possibility of using a straightforward synthesis with a nature-inspired compound for most probably a common, simple, and budget electroactive nanomaterial for electrochemical research. The advantage of the presented sensor was conducting them at low potentials, which increases the selectivity of the system.

It is also significant that when this work was undertaken, there were no existing literature reports on this type of biomolecular corona with tests on real samples (human blood, human serum) and study of the influence of interference. 

## 2. Results and Discuss

### 2.1. Morphological Characterization

**Figure 1:**
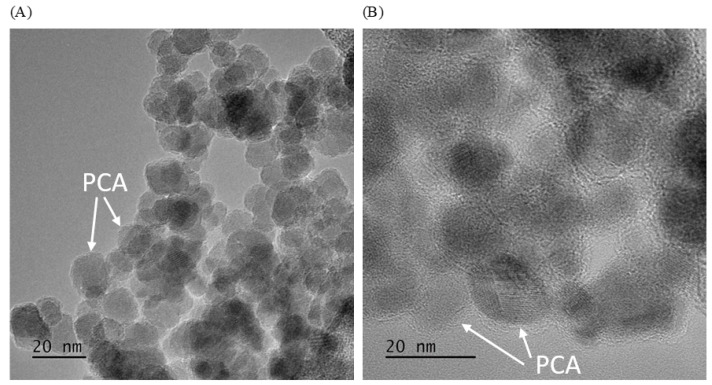
HRTEM images (**A**); in increased magnification (**B**) of Fe_3_O_4_@PCA.

To characterize the morphology of the material nanoparticles after the poly(caffeic acid) coating process, a high-resolution transmission electron microscopy (HRTEM) study was performed. The study was carried out on the Fe_3_O_4_@PCA nanomaterial, and Figure 1 shows the image obtained. As Figure 1 shows, magnetite nanoparticles with a diameter of 8–12 nm obtained by the co-precipitation method were evenly covered with a poly(caffeic acid) coating with a diameter of 3–4 nm.

**Figure 2:**
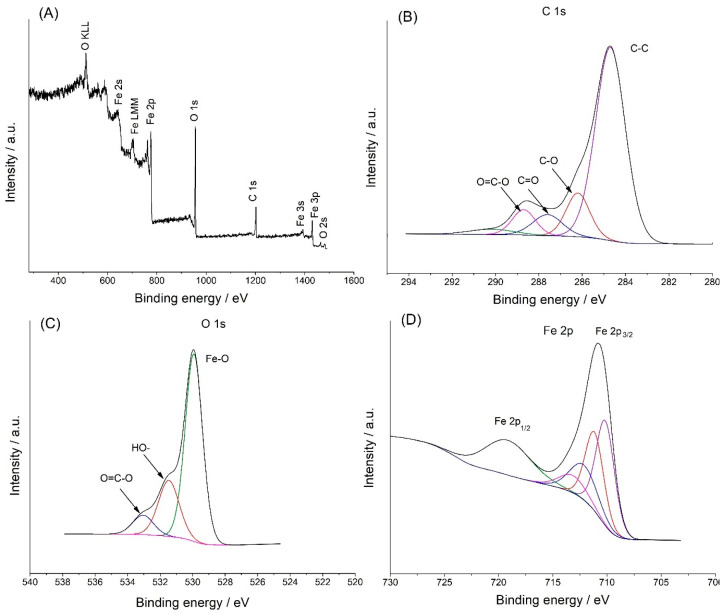
XPS spectrum of the hybrid Fe_3_O_4_@PCA (**A**); XPS results of C 1s (**B**); O 1s (**C**); Fe 2p (**D**).

The X-ray photoelectron (XPS) measurements were used to evaluate the oxidation states and elemental composition. Figure 2A shows the XPS survey spectrum of Fe_3_O_4_@PCA, which demonstrated the existence of C, O, and Fe elements, which were excepted for the final product. Figure 2B presents the deconvoluted XPS spectrum of C 1s, which demonstrated fitting peaks at binding energies of 284.6, 286.2, 287.5, 288.6, which can be related to C-C, C-O, C=O, and O=C-O chemical bonds, respectively [17,18]. Three oxygen contributions, 529.80, 530.9, and 531.31 eV (Figure 2C), can be assigned to Fe-O, -OH, and O=C-O groups, respectively [18,19]. The deconvoluted XPS spectrum for Fe 1s XPS presented the fitted peaks at 711.1 eV (Fe 2p^3/2^) and 720.2 eV (Fe 2p^1/2^), confirming the successful functionalization of magnetite with poly(caffeic acid) [18]. 

**Figure 3:**
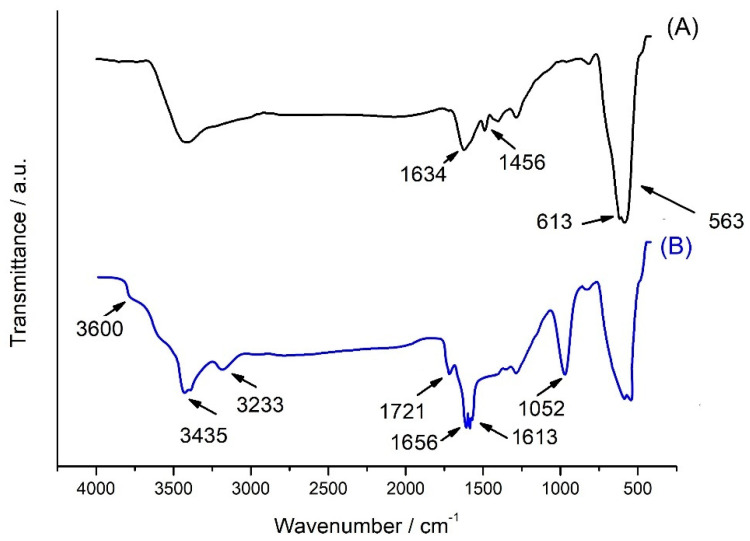
FT-IR spectra of Fe_3_O_4_ (A); Fe_3_O_4_@PCA (B).

Physicochemical analysis for the presence of magnetite and magnetite@poly(caffeic acid) was proven using the FTIR data (Figure 3). Both curves a and b show a clear peak at 563 cm^−1^, corresponding to the Fe-O bond. This was confirmed by the formation of magnetite nanoparticles in the case of magnetite as a core [20]. Both spectra (a and b) show the 3400 cm^−1^ absorption peak, which is present because of the O–H bands in the structure [20]. Observed 1656 and 1613 cm^−1^ peaks were assigned to the C=O and C=C vibrations, respectively, characteristic for PCA [21].

### 2.2. Immobilization of Glucose Oxidase (GOx)

To immobilize the enzyme–glucose oxidase, adsorption immobilization was performed. The method was optimized using two nanoplatforms, Fe_3_O_4_ NPs and Fe_3_O_4_@PCA. The process was carried out at different times (1, 2, 5, 10, 24, 48 h). As Table 1 shows, the highest amount of the enzyme (determined by the Bradford method) was immobilized within 24 h for both platforms. Within 24 h, 24.5 mg and 32 mg of the enzyme were immobilized per g of the matrix for Fe_3_O_4_ and Fe_3_O_4_@PCA, respectively. Above 24 h, a decrease in the amount of immobilized enzyme was observed, which may be due to the washing out of the enzyme.

For comparison, the efficiency of GOx immobilization reported by Lee and co-authors on the surface of single-walled carbon nanotubes (SWNTs) was 20.7 mg g^−1^ [22]. In our previous studies on Fe_3_O_4_@PDA and Fe_3_O_4_@βCD nanoplatforms, we managed to immobilize 36.3 mg g^−1^ and 47.6 mg g^−1^ of glucose oxidase, respectively [23]. The efficiency of immobilization by the Bradford method was also determined on the Fe_3_O_4_@Lig/PDA material with an efficiency of 29.44 mg g^−1^ of the attached enzyme [24].

**Figure 4:**
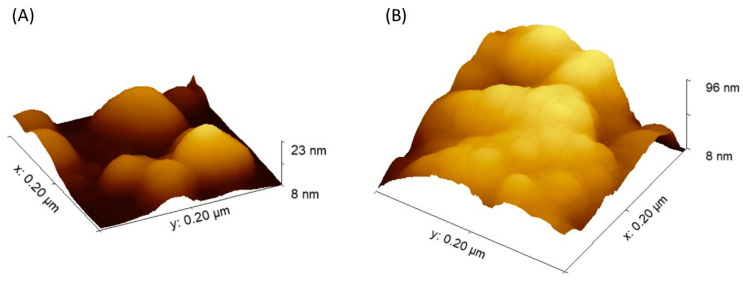
AFM images of Fe_3_O_4_@PCA nanomaterial before (**A**); and after GOx immobilization (**B**).

Another method confirming the immobilization process’s effectiveness and showing the material’s morphology after the immobilization process was atomic force microscopy (AFM). Figure S1 (see Supplementary Materials) and Figure 4 show the atomic force microscopy images (2D and 3D) for the Fe_3_O_4_@PCA matrix before and after immobilization, respectively. Significant changes in the material after immobilization can be observed between these surfaces. The more than fourfold increase in the Z parameter may indirectly confirm the successful attachment of GOx to the surface of the nanomaterial matrix.

Based on the AFM images, Figure S2 (see Supplementary Materials) shows the height profiles for the nanomaterial before and after immobilization were determined. To characterize the matrices, the roughness coefficient (Sa) of 9.9 ± 0.3 and 6.5 ± 0.2 was determined for Fe_3_O_4_ and Fe_3_O_4_@PCA, respectively. The roughness parameter was reduced due to the enzyme’s smoothing effect on the surface.

### 2.3. Electrochemical Tests of SPE/Fe_3_O_4_@PCA-GOx Electrode

**Figure 5:**
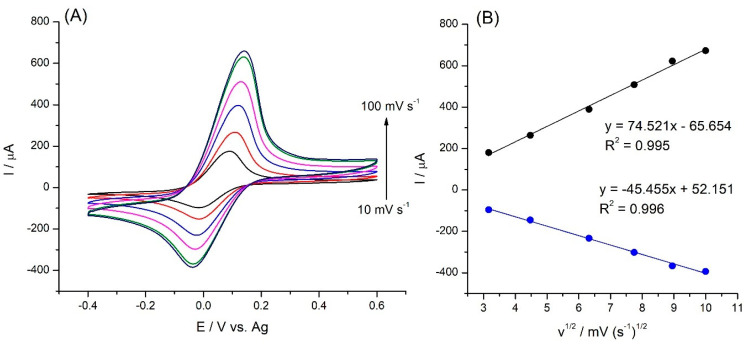
CVs of SPE/Fe_3_O_4_@PCA-GOx at various scan rates (10–100 mV s^-1^) (**A**); plots of anodic and cathodic peak currents vs. square root of scan rate (**B**).

The influence of the scanning rate on the SPE/Fe_3_O_4_@PCA-GOx response of the biosensor was examined by cyclic voltammetry (CV). A non-conductive protein coating on an enzyme serves as an insulating barrier, effectively blocking the transfer of electrons to the active electrode’s surface. This unique characteristic presents a compelling necessity for conducting research in the presence of an external mediator [25]. Figure 5A shows the change in the response of the SPE/Fe_3_O_4_@PCA-GOx biosensor in the presence of hydroxy(methyl ferrocene) as a mediator. 

Figure 5B shows a linear increase between the peak current and the square root of the scan rate, indicating that the reaction is a diffusion-controlled process. 

**Figure 6:**
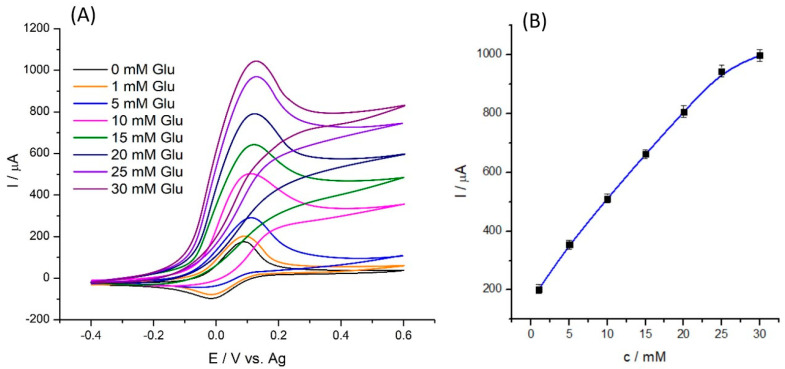
Cyclic voltammograms in PBS solution containing 10 mM HFc and various concentrations of glucose at scan rate 10 mV s^−1^ (**A**); current (I/μA) vs. glucose concentration (**B**) (mM) (n = 3).

Using cyclic voltammetry tests, the electrocatalytic effect of the SPE/Fe_3_O_4_@PCA-GOx biosensor was investigated, and Figure 6 shows the results. Figure 6A shows visible redox peaks for the oxidation and reduction processes of the mediator at 0.09 and −0.01 V, respectively. The shift of potentials in relation to the characteristic for the mediator results from the files are characteristic for poly(caffeic acid) rich in catechol groups. The resulting peaks were formed due to oxidation and reduction of the mediator used—hydroxy(methyl ferrocene). After adding glucose to the PBS solution and the mediator, an increase in the oxidation peak and a decrease in the reduction peak were observed, indicating that the enzyme-catalyzed reaction of the immobilized GOx toward glucose was taking place at the SPE/Fe_3_O_4_@PCA-GOx electrode. As a result of the reactions, the reduced HFc is converted into the catalytically active form—HFc^+^. In the next step, FADH_2_ and HFc^+^ exchange electrons, producing FAD regeneration and HFc production. As Figure 6A shows, the biosensor response (increase in current) is proportional to the amount of added glucose. Figure 6B shows the response curve which follows a calibration curve presenting the linearity in the initial stages, approaching saturation for higher substrate concentration characteristics for the enzyme [26].

**Figure 7:**
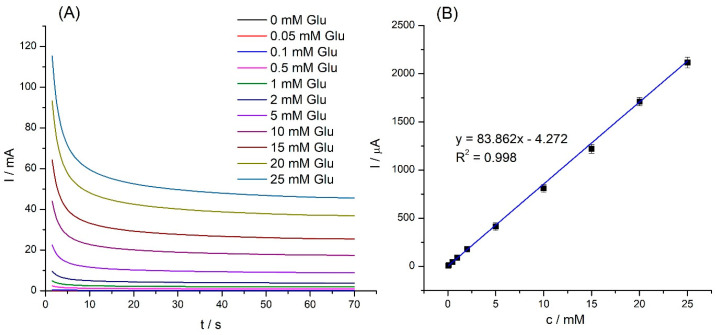
The amperometric responses of SPE/Fe_3_O_4_@PCA for various glucose concentrations (0.05–25 mM) in 50 mM PBS at +0.1 V (**A**); the corresponding calibration curve of current response vs. glucose concentration (**B**) (n = 3).

To verify the correct operation of the biosensor and the effectiveness of glucose detection, amperometric tests were carried out. The assays were carried out in a solution of 50 mM PBS (pH 7.4) together with a 10 mM mediator hydroxy(methyl ferrocene) at a potential of 0.1 V at various concentrations of glucose (0–25 mM). Figure 7 shows the obtained amperometric curves.

As Figure 7A shows, the current response of the SPE/Fe_3_O_4_@PCA-GOx biosensor increased with increased glucose concentration. Figure 7B shows the calibration plot of the response current to different glucose concentrations (0.05–25 mM). The current was read after 60 s of measurements and then the baseline was subtracted. The proposed biosensor was characterized with a linear range from 0.05 to 25.0 mM of glucose, with a sensitivity of 1198.0 μA mM^−1^ cm^−2^, a calculated limit of detection (LOD) of 5.23 μM, and a limit of quantification (LOQ) of 15.85 μM. 

Table 2 presents a comparison of the constructed SPE/Fe_3_O_4_@PCA-GOx biosensor with other glucose biosensors based on glucose oxidase (GOx).

### 2.4. Interferents Tests

**Figure 8:**
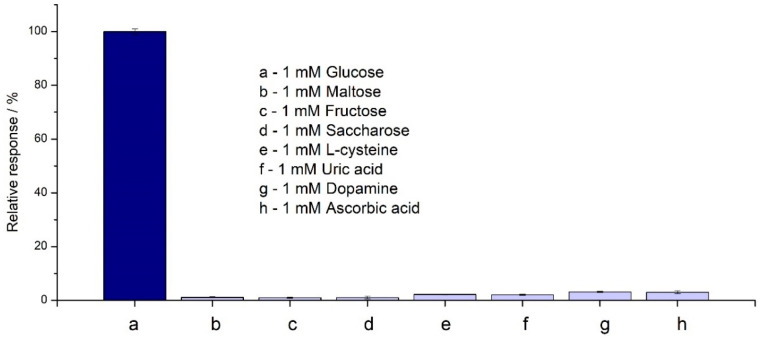
Interferents tests of the biosensor in 50 mM PBS at +0.1 V (n = 3).

Selectivity is one of the most important features of the biosensor, which has a significant impact on the final measurement of the detector. This factor is affected by two types of reactions: enzyme-analyte and selective electrochemical measurement. Ascorbic acid, uric acid, and dopamine are biomolecules that interrupt high polarization voltage in bodily fluids where they oxidize and produce incorrect signals [34]. To check the influence of the interferents on the proposed SPE/Fe_3_O_4_@PCA-GOx system, tests of the individual interferents (without the presence of glucose), i.e., maltose, saccharose, fructose, L-cysteine, uric acid, dopamine, and ascorbic acid, were performed (Figure S3, see Supplementary Materials). The responses obtained were then compared to glucose responses and are shown as relative responses, as Figure 8 shows. 

Figure 8 shows that the interfering response was negligible in the range of 0.88% (fructose) to 3.01% (dopamine). The selectivity for various monosaccharides results from the GOx used. This indicates the high selectivity of the used enzyme–glucose oxidase. However, using a low potential (0.1 V) made it possible to eliminate the oxidation of electroactive compounds, like dopamine or ascorbic acid, which is an advantage when using higher potentials.

To compare the selectivity of the biosensors, for concentrations 10 times lower than glucose interferents, the effect was up to 3.85% presented by Chen et al. on the proposed Ni(OH)_2_/ECF system [35]. In the work presented by Li et al., the proposed PtNWA/AuNPs/GOx sensor showed a response to uric acid (5.57%) with the same content of glucose [36]. As presented by Zhang et al., selectivity measurements were conducted at 0.75 V with the Au/OPPy/AuNPs/GOx/Nafion electrode. The presented responses relative to glucose were 5.4% for ascorbic acid and 4.8% for urea [37]. This shows that the results obtained based on the Fe_3_O_4_@PCA-GOx electrode presented high selectivity.

### 2.5. Optimization Tests

The effects of experimental conditions (various pH and temperature) on the glucose SPE/Fe_3_O_4_@PCA-GOx biosensor response were investigated to determine optimal conditions. The pH dependence of the biosensor was tested in 1 mM glucose solution in the pH range of 3–10 (Figure S4A, see Supplementary Materials). The biosensor showed an optimal response, characterized by the highest currents at pH 7.4. Immobilization of the enzyme affects the nanomaterial influence on the protein conformation of and changes in the optimum operating pH [38].

Thermal stability is an important practical parameter in the application of biosensors due to the susceptibility of enzymes to denaturation [38]. The stability of the biosensor was tested in the presence of 1 mM glucose in the temperature range from 10 to 50 °C; however, the current increased up to 40 °C, and above this temperature the signal decreased (Figure S4B, see Supplementary Materials). This may be caused by partial denaturation of the enzyme [38]. However, due to the widespread use of this type of biosensor, room temperature is optimal for testing and was chosen for further research.

Research was carried out to check the size of the sensor’s response after immobilization (Figure S4C, see Supplementary Materials). For this purpose, the same time range was examined as in the Bradford method. As can be seen, the highest current response was measured for 24 h of immobilization, which led to this system being characterized by the highest sensitivity. Different sensitivities in enzymatic electrochemical biosensors can significantly impact the limit of detection (LOD) because sensitivity determines how effectively the sensor can detect even minor changes in the concentration of the target analyte. Higher sensitivity enables the sensor to produce a more substantial and distinguishable signal response in the presence of lower analyte concentrations, resulting in a better signal-to-noise ratio and enhanced accuracy.

Various values (from 0.5 to 20 mM) were examined to determine the optimal mediator concentration, and Figure S4D (see Supplementary Materials) displays the results. As the sensor response somewhat increases above 10 mM, in the testing that followed, this concentration was used [38].

### 2.6. Real Sample Analysis

To determine the biosensor detection in real solutions, glucose detection in human serum and human blood was carried out. This enabled the verification of the operation in a real solution containing interfering agents. The concentration of glucose in the tested solutions was also checked using a chemical analyzer (spectrophotometric method) in the hospital. The tests were performed in triplicate, and the results are presented as the average in Table 3.

The glucose content was tested in three human serum samples with different concentrations (2.8 mM; 6.9 mM; 9.2 mM). The obtained data showed that the proposed biosensor recovered in the range of 97.9 to 99.1%. In the next step, the constructed biosensor was used to test human blood samples (3.2 mM; 6.3 mM; 14.7 mM glucose). The obtained results allowed for recovery in the 98.2 to 98.6% range. Moreover, the obtained results align with the concentrations recommended by the World Health Organization (WHO) [39].

### 2.7. Time Stability and Reproducibility of the SPE/Fe_3_O_4_@PCA-GOx Biosensor

Constructing a time-stable glucose biosensor based on glucose oxidase (GOx) requires the enzyme to maintain a functional level of catalytic activity. Therefore, the optimal immobilization and control of the biosensor’s stability over time are essential [40].

**Figure 9:**
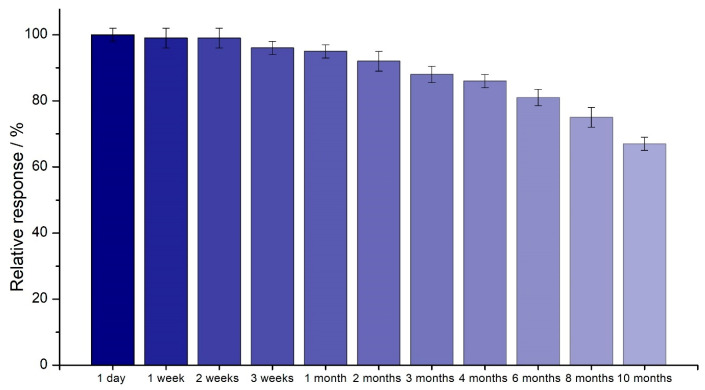
Comparison of the long-term stability as a relative response to 2 mM glucose for the SPE/Fe_3_O_4_@PCA-GOx biosensor (n = 3).

For long-term stability, the proposed SPE/Fe_3_O_4_@PCA-GOx biosensor was tested. The study was conducted using amperometry at a potential of 0.1 V. The duration of the study was 10 months. Between measurements, the electrode was stored dry in the fridge at 4 °C. The test was repeated 3 times. Figure 9 shows the relative response’s dependence on the biosensor’s viability. 

Table 4 presents a comparison of the stability over time of the developed biosensor with other GOx-based glucose biosensors.

The biosensor’s reproducibility refers to its ability to generate the same results under identical testing conditions. The transducer and electronics in a biosensor are precise and accurate, which defines reproducibility (inter-day) [45]. The reproducibility assay value for constructed SPE/Fe_3_O_4_@PCA-GOx electrode was 1.1%, which characterizes the detection procedure as repeatable. 

The same SPE/Fe_3_O_4_@PCA-GOx electrode was used eight times at amperometric measurements (2 mM glucose, 0.1 V), and output currents were measured to check the repeatability of the biosensor. These measurements with the same electrode presented an RSD value of 2.7%, indicating high repeatability (intra-day).

## 3. Materials and Methods

### 3.1. Chemicals and Materials

Iron(II) chloride tetrahydrate (FeCl_2_·4H_2_O), iron(III) chloride hexahydrate (FeCl_3_ · 6H_2_O), and ammonia solution (25%) to magnetite nanoparticles were obtained from Merck, Poznan, Poland. Caffeic acid (≥98.0%), sodium periodate (≥98.0%), and tris (hydroxymethyl)aminomethane (TRIS) were provided from Merck, Poznan, Poland. Glucose Oxidase from Aspergillus niger (protein content 65–85%, molecular weight 160 kDa) and citric buffer were purchased from Merck, Poland. The phosphate buffer saline solution (PBS; 50 mM; pH 7.4) was prepared using K_2_HPO_4_ and KH_2_PO_4_ (POCH, Gliwice, Poland). (Hydroxymethyl)ferrocene (HFc) was purchased from Alfa Aesar (Ward Hill, MA, USA). The detection analyte α-D-glucose was purchased from Merck, Poland. The tested interferents, i.e., maltose, sucrose, fructose, uric acid, ascorbic acid, dopamine, and L-cysteine, were obtained from Merck, Poznan, Poland. The human serum (male, group AB) was purchased from Merck, Poznan, Poland. Human blood samples were provided by Bio-Rad (Hercules, CA, USA).

### 3.2. Synthesis of Fe_3_O_4_@PCA-GOx Biomolecular Corona

To obtain magnetite nanoparticles by co-precipitation, 1.72 g of FeCl_3_·6H_2_O (11.09 mM) and 0.86 g of FeCl_2_·4H_2_O (7.55 mM) were added to 50 mL of Milli-Q^®^ water. Then, the source was heated to 90 °C, and the nanoparticles were obtained in a nitrogen gas environment. Ten mL of a 25% aqueous ammonia solution was added to the mixture, and then, after 30 min, the solution was cooled to ambient temperature. The proposed conditions allowed the manipulation of the size and shape of nanoparticles.

The coating of magnetite nanoparticles with poly(caffeic acid) was carried out by chemical synthesis. Fifty mg of the obtained nanoparticles was added to 100 mL of TRIS buffer (pH 8.5, 10 mM) and then subjected to ultrasound. Then, 50 mg of caffeic acid and 60 mg of NaIO_4_ were added. The process was carried out without access to oxygen for 24 h under stirring conditions. Immobilization of glucose oxidase (GOx) on the surface of Fe_3_O_4_@PCA material was carried out by the adsorption method. For this purpose, 5 mg of Fe_3_O_4_@PCA and 0.5 mg of GOx were dissolved in 1 mL of citric buffer (10 mM, pH 5.5). The process was carried out for a different time (1–48 h) under constant mixing conditions.

### 3.3. Fabrication of SPE/Fe_3_O_4_@PCA-GOx Electrode

To construct an electrochemical glucose biosensor, the surface of the SPE electrode (with carbon working electrode, auxiliary electrode made by carbon, and silver reference electrode) was modified using the obtained nanomaterial Fe_3_O_4_@PCA-GOx (4.89 mg mL^−1^). After dropping, the material was allowed to dry at 4 °C. 

### 3.4. Physicochemical Analysis

High-resolution transmission electron microscopy (HRTEM) testing was performed with a Jeol analyzer (Jeol ARM 200F (Jeol, Akishima, Tokyo, Japan); resolution of 0.63 Å and a maximum acceleration of 200 kV). Fourier infrared spectroscopy (FTIR) for functional group characterization was performed using a Vertex 70 spectrometer. The pellets were prepared by mixing 2 mg of the material (Fe_3_O_4_, Fe_3_O_4_@PCA, Fe_3_O_4_@PCA-GOx, and GOx) with 250 mg of anhydrous Potassium Bromide (KBr). The AFM (Atomic Force Microscope, Park NX10) was used to analyze the material’s morphology. Zeta potential (ζ) and polydispersity index (PDI) value studies were conducted with a Zetasizer Nano ZS to check the stability and homogeneity of the materials. The research was conducted in the range of 0.6–6000 nm. With the use of the Prevac, UHV multi-chamber analytical system, the XPS investigation was carried out. A VG Scienta SAX 100 X-ray amp with an aluminum anode and a VG Scienta XM 780 monochromator served as the radiation source, which emitted radiation with the Al K characteristic line and an energy of 1486.7 eV.

### 3.5. Electrochemical Study

Electrochemical measurements were carried out to characterize the obtained biosensor. The studies were carried out using the μ-Autolab III potentiostat/galvanostat (ECO Chemie, Netherlands). The tests were carried out using an SPE electrode supplied by Palmsens (Houten, Netherlands). The tests were carried out in (10 mM; pH 7.4) and 10 mM mediator (hydroxymethyl) ferrocene (HFc). Cyclic voltammetry studies were conducted in the potential range of −0.4 to 0.6 V, using 10 mV s^−1^ as the scanning rate. Interferent studies and optimization were carried out using amperometric methods. All amperometric tests were carried out at a constant potential of 0.1 V. Stability over time was carried out for 10 months. Between measurements, the electrode was stored at 4 °C. Tests on real solutions (human blood, human serum) were carried out using the standard addition method.

## 4. Conclusions

In this work, the poly(caffeic acid)@magnetite nanomaterial was obtained and applied for glucose level monitoring. The biomolecular corona-based hybrid nanomaterial was used for the immobilization of glucose oxidase. The miniaturization of the system was carried out by modifying the screen-printed (SPE) electrode. The obtained biosensor was used to measure glucose in a wide range of concentrations, from hypoglycemia to diabetes, in accordance with WHO guidelines.

The most significant advantages of the proposed biosensor are low electrochemical potential, time stability, sensitivity, selectivity, and reproducibility. The proposed biosensor was also tested with real solutions, like human serum and human blood. The SPE/Fe_3_O_4_@PCA-GOx biosensor unveils an opportunity to be successfully used in glucose measurements.

## Figures and Tables

**Table 1:** Quantities of enzyme immobilized on Fe_3_O_4_, and Fe_3_O_4_@PCA from glucose oxidase solution at various times (n = 3).

Time/h	Fe_3_O_4_ NPs/mg g^−1^	Fe_3_O_4_@PCA/mg g^−1^
1	1.4 ± 0.2	4.3 ± 0.3
2	3.2 ± 0.4	10.2 ± 0.2
5	6.2 ± 0.3	18.3 ± 0.4
10	10.2 ± 0.3	24.2 ± 0.2
24	17.3 ± 0.2	38.1 ± 0.3
48	15.5 ± 0.4	30.2 ± 0.5

**Table 2:** Comparison of the analytical performance of various glucose GOx-based biosensors.

Electrode	Sensitivity (μA mM^−1^ cm^−2^)	Limit of Detection (μM)	Linear Range (mM)	References
Fe_3_O_4_@PNE-GOx	97.30	6.10	0.2–24.0	[4]
GOx-SiO_2_/Lig/Fc/CPE	11.0	145.0	0.5–9.0	[27]
GOx_EPC_-Den_Au_/CC	72.45	6.70	0.02–31.7	[25]
RA–PANI/CS–GOx	22.10	2.77	0.01–1.09	[28]
PANI-TT-GOx	23.57	1.0	0.005–5.0	[29]
TiO_2_NWc/GOx	58.90	8.7	0.0–2.0	[30]
Fe_3_O_4_−CS−CD/MWCNTs/GOx	23.59	19.3	0.04–1.04	[31]
TiO_2_/Au/GOx	16.86	0.83	0.01–3.0	[32]
CNS-Nafion-GOx	7.31	39.1	0.08–2.04	[33]
**Fe_3_O_4_@PCA-GOx**	**1198.0**	**5.23**	**0.05–25.0**	**This work**

**Table 3:** Detection of glucose in human serum and human blood samples by the SPE/Fe_3_O_4_@PCA-GOx sensor.

Sample	Glucose Concentration/mM	SPE/Fe_3_O_4_@PCA-GOx	
		Find/mM	Recovery/%
human serum	2.8	2.74 ± 0.05	97.9 ± 1.79
	6.9	6.84 ± 0.05	99.1 ± 0.72
	9.2	9.09 ± 0.10	98.8 ± 1.08
human blood	3.2	3.14 ± 0.04	98.2 ± 1.25
	6.3	6.21 ± 0.06	98.6 ± 0.95
	14.7	14.43 ± 0.05	98.2 ± 0.34

**Table 4:** Comparison of glucose biosensor systems’ long-term storage stability.

Biosensor System	Maintained Storage Stability (%)	Storage Time (Days)	Reference
SPE/Fe_3_O_4_@PNE-GOx	75.1	140	[4]
Ppy/GOx/GR	29.7	35	[41]
CHI-GOx/APTES/dPIn	76.7	28	[42]
Go/Co/chitosan-GOx	70.0	14	[43]
GC/MWCNT/Fe_3_O_4_/PDA/β-CD-GOx	63.0	210	[23]
CNT/PEI/GOx	66.7	120	[44]
**SPE/Fe_3_O_4_@PCA-GOx**	**67.5**	**300**	**This work**

## Data Availability

All data used to write this manuscript can be found in the manuscript or Supplementary Material.

## Supplementary Materials

The following supporting information can be downloaded at: https://www.mdpi.com/article/10.3390/molecules28217281/s1, Figure S1: AFM 2D images of Fe_3_O_4_@PCA nanomaterial before (A); and after GOx immobilization (B); Figure S2: Height profile plots for Fe_3_O_4_@PCA (A); and Fe_3_O_4_@PCA-GOx (B); Figure S3: Amperometric response of the GC/Fe_3_O_4_@PCA-GOx electrode with the addition of 1 mM glucose (A) and subsequent additions of 1 M maltose (B), 1 mM fructose (C), 1 mM saccharose (D), 1 mM L-cysteine (E), 1 mM uric acid (F), 0.1 mM dopamine (G), and 1 mM ascorbic acid (H) at +0.1 V. Figure S4: The effect of pH (A); temperature (B) on the response of the SPE/Fe_3_O_4_@PCA-GOx biosensor (1 mM of glucose, at +0.1 V), (n = 3).
