# Supplementary material for: Nature-Inspired Biomolecular Corona Based on Poly(caffeic acid) as a Low Potential and Time-Stable Glucose Biosensor
Source: Molecules. 2023 Oct 26;28(21):7281. doi: 10.3390/molecules28217281 (PMC10649105; doi:10.3390/molecules28217281)
Supplement: Supplementary file 1 [file molecules-28-07281-s001.zip › molecules-2647041-SI.pdf]

Nature-inspired biomolecular corona-based on poly(caffeic acid)

as

a low potential and time-stable glucose biosensor

Maria Kuznowicz, Artur Jędrzak*, Teofil Jesionowski*

[1]Institute of Chemical Technology and Engineering, Faculty of Chemical Technology, Poznan University of Technology, Berdychowo 4, PL-60965 Poznan, Poland;
*Corresponding authors: artur.jedrzak@put.poznan.pl and teofil.jesionowski@put.poznan.pl

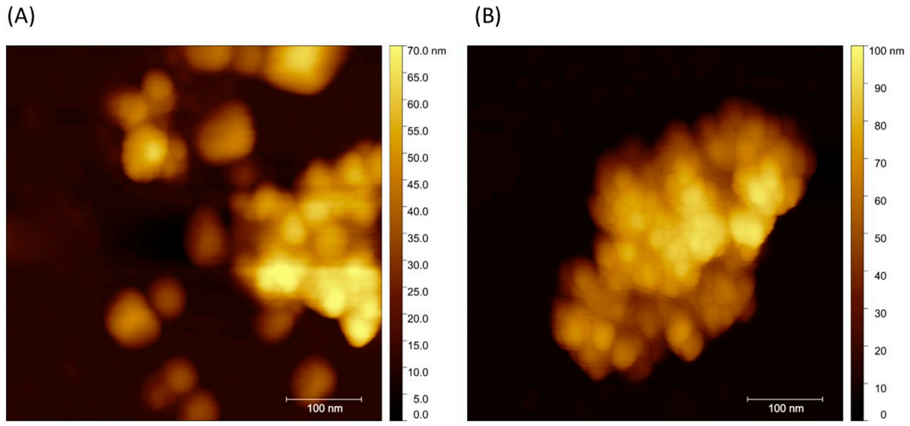

Figure S1. AFM 2D images of Fe_3O_4@PCA nanomaterial before (A); and after GOx immobilization (B)

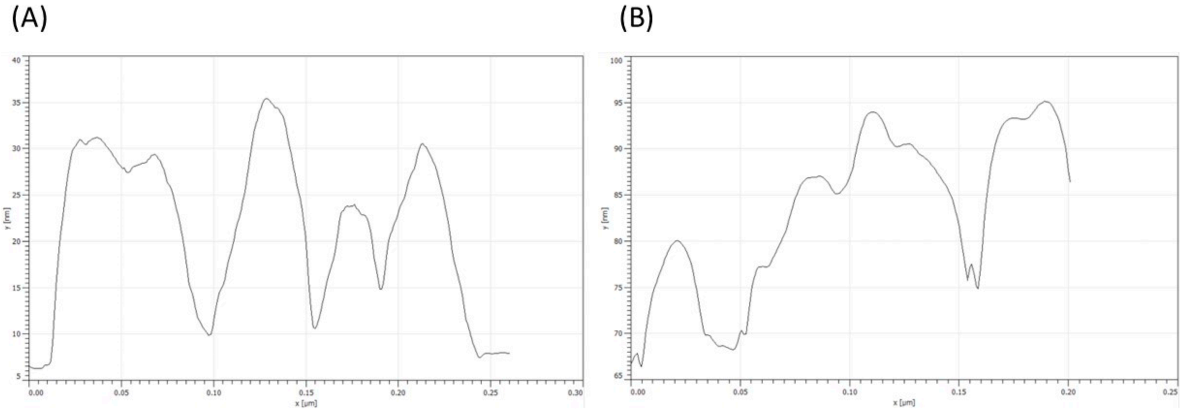

Figure S2. Height profile plots for Fe_3O_4@PCA (A); and Fe_3O_4@PCA-GOx (B).

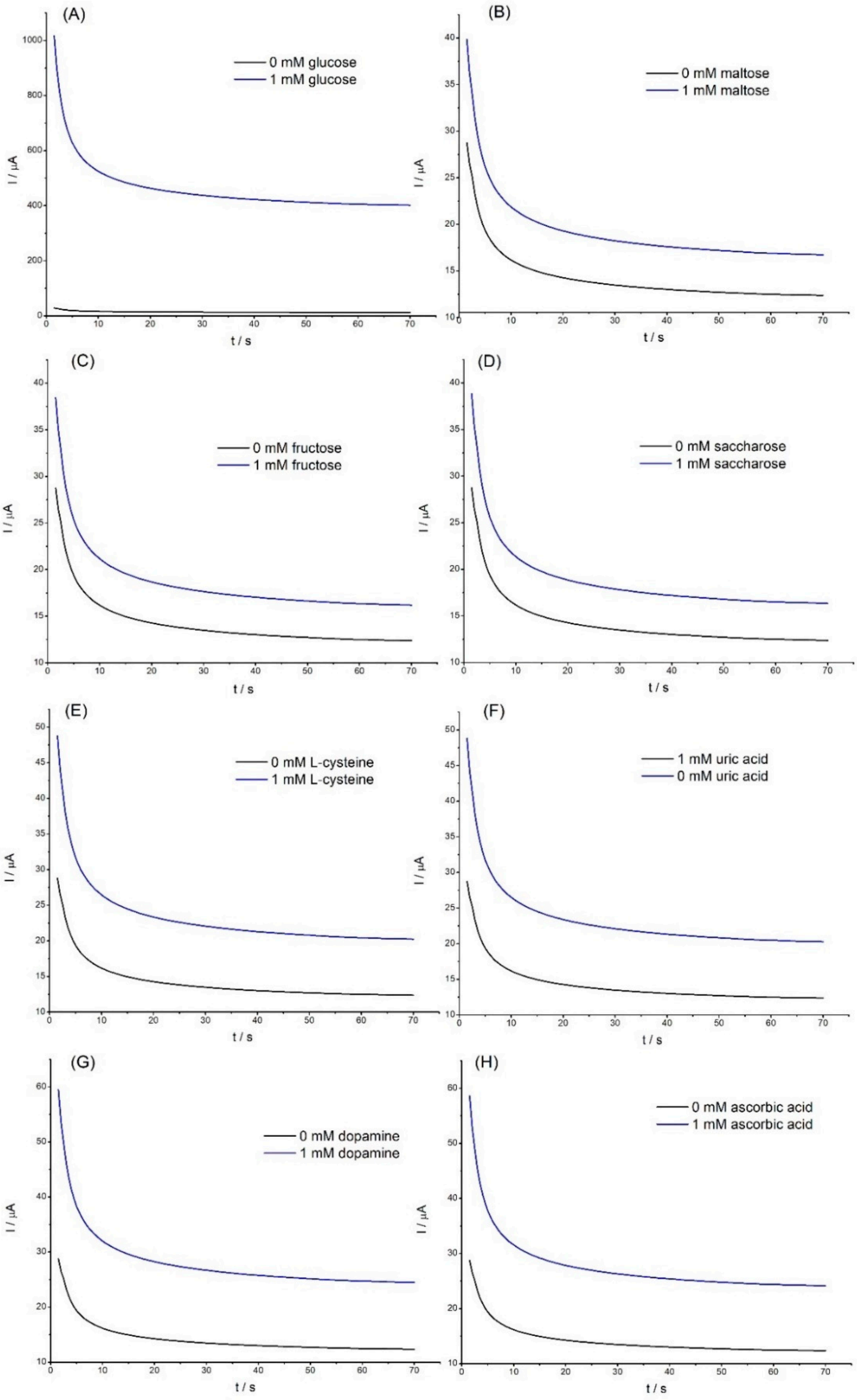

Figure S3. Amperometric response of the GC/Fe_3O_4@PCA-GOx electrode with the addition of 1 mM glucose (A) and subsequent additions of 1 M maltose (B), 1 mM fructose (C), 1 mM saccharose (D), 1 mM L-cysteine (E), 1 mM uric acid (F), 0.1 mM dopamine (G), and 1 mM ascorbic acid (H) at +0.1 V.

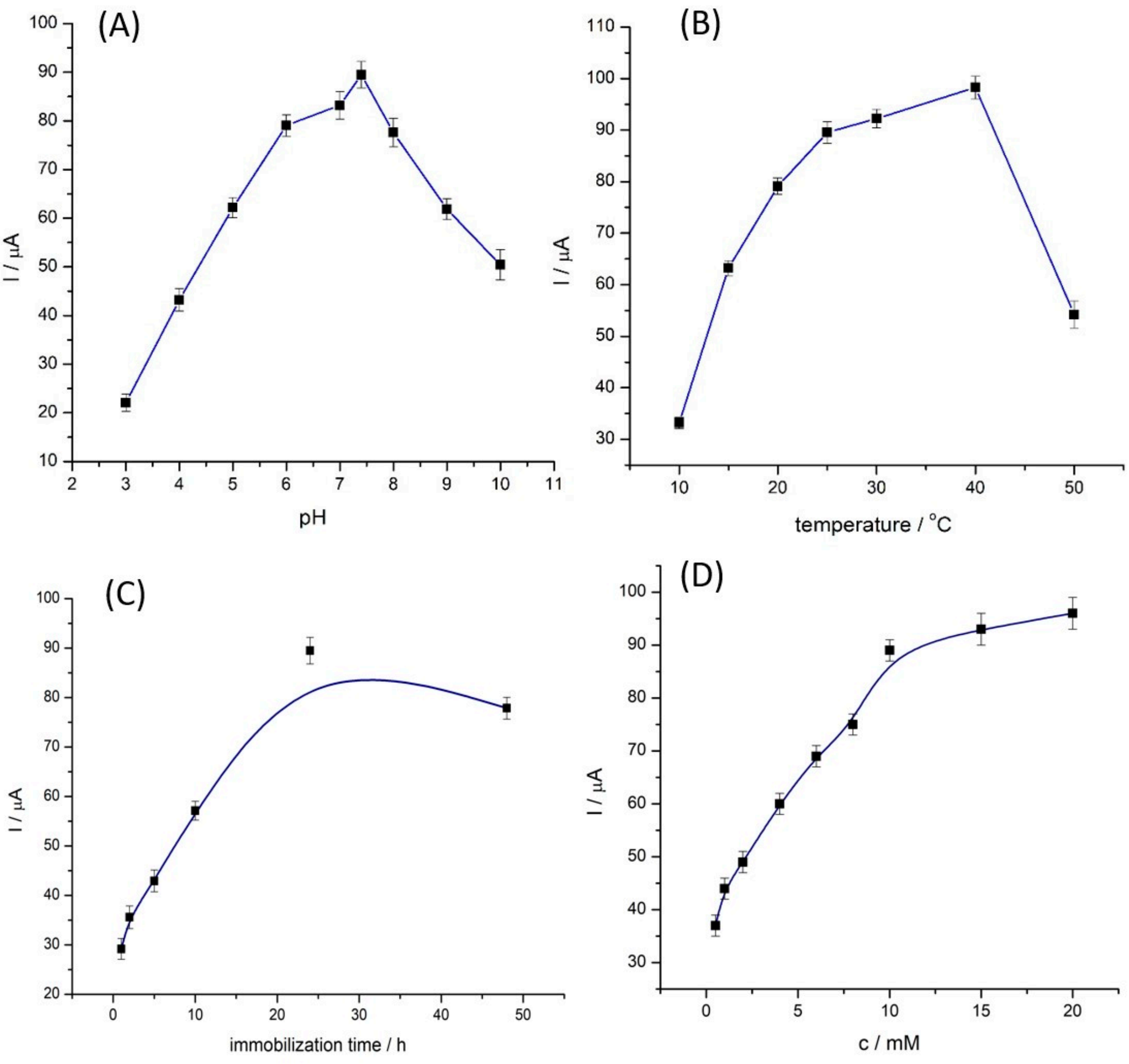

Figure S4. The effect of pH (A); temperature (B); immobilization time (C); mediator concentration (D), on the response of the SPE/Fe_3O_4@PCA-GOx biosensor (1 mM of glucose, at +0.1 V), (n=3)
